# Supplementary material for: The Discontinuous Elevational Distribution of an Ungulate at the Regional Scale: Implications for Speciation and Conservation
Source: Animals (Basel). 2021 Dec 15;11(12):3565. doi: 10.3390/ani11123565 (PMC8697900; doi:10.3390/ani11123565)
Supplement: Supplementary file 1 [file animals-11-03565-s001.zip › animals-1444872-supplementary.pptx]

## Slide 1
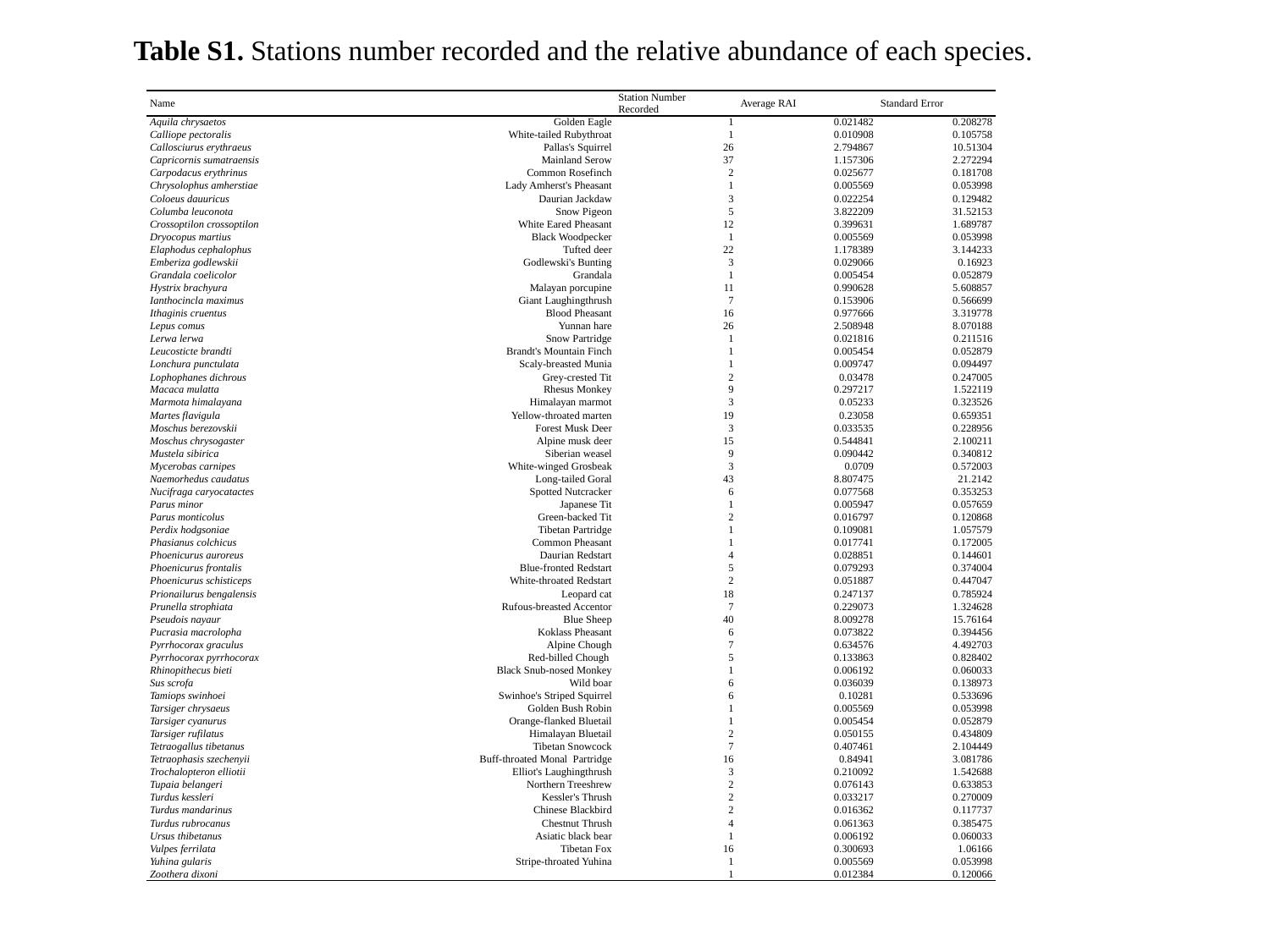

Table S1. Stations number recorded and the relative abundance of each species.
| Name | | | Station Number Recorded | Average RAI | Standard Error |
| --- | --- | --- | --- | --- | --- |
| Aquila chrysaetos | | Golden Eagle | 1 | 0.021482 | 0.208278 |
| Calliope pectoralis | | White-tailed Rubythroat | 1 | 0.010908 | 0.105758 |
| Callosciurus erythraeus | | Pallas's Squirrel | 26 | 2.794867 | 10.51304 |
| Capricornis sumatraensis | | Mainland Serow | 37 | 1.157306 | 2.272294 |
| Carpodacus erythrinus | | Common Rosefinch | 2 | 0.025677 | 0.181708 |
| Chrysolophus amherstiae | | Lady Amherst's Pheasant | 1 | 0.005569 | 0.053998 |
| Coloeus dauuricus | | Daurian Jackdaw | 3 | 0.022254 | 0.129482 |
| Columba leuconota | | Snow Pigeon | 5 | 3.822209 | 31.52153 |
| Crossoptilon crossoptilon | | White Eared Pheasant | 12 | 0.399631 | 1.689787 |
| Dryocopus martius | | Black Woodpecker | 1 | 0.005569 | 0.053998 |
| Elaphodus cephalophus | | Tufted deer | 22 | 1.178389 | 3.144233 |
| Emberiza godlewskii | | Godlewski's Bunting | 3 | 0.029066 | 0.16923 |
| Grandala coelicolor | | Grandala | 1 | 0.005454 | 0.052879 |
| Hystrix brachyura | | Malayan porcupine | 11 | 0.990628 | 5.608857 |
| Ianthocincla maximus | | Giant Laughingthrush | 7 | 0.153906 | 0.566699 |
| Ithaginis cruentus | | Blood Pheasant | 16 | 0.977666 | 3.319778 |
| Lepus comus | | Yunnan hare | 26 | 2.508948 | 8.070188 |
| Lerwa lerwa | | Snow Partridge | 1 | 0.021816 | 0.211516 |
| Leucosticte brandti | | Brandt's Mountain Finch | 1 | 0.005454 | 0.052879 |
| Lonchura punctulata | | Scaly-breasted Munia | 1 | 0.009747 | 0.094497 |
| Lophophanes dichrous | | Grey-crested Tit | 2 | 0.03478 | 0.247005 |
| Macaca mulatta | | Rhesus Monkey | 9 | 0.297217 | 1.522119 |
| Marmota himalayana | | Himalayan marmot | 3 | 0.05233 | 0.323526 |
| Martes flavigula | | Yellow-throated marten | 19 | 0.23058 | 0.659351 |
| Moschus berezovskii | | Forest Musk Deer | 3 | 0.033535 | 0.228956 |
| Moschus chrysogaster | | Alpine musk deer | 15 | 0.544841 | 2.100211 |
| Mustela sibirica | | Siberian weasel | 9 | 0.090442 | 0.340812 |
| Mycerobas carnipes | | White-winged Grosbeak | 3 | 0.0709 | 0.572003 |
| Naemorhedus caudatus | | Long-tailed Goral | 43 | 8.807475 | 21.2142 |
| Nucifraga caryocatactes | | Spotted Nutcracker | 6 | 0.077568 | 0.353253 |
| Parus minor | | Japanese Tit | 1 | 0.005947 | 0.057659 |
| Parus monticolus | | Green-backed Tit | 2 | 0.016797 | 0.120868 |
| Perdix hodgsoniae | | Tibetan Partridge | 1 | 0.109081 | 1.057579 |
| Phasianus colchicus | | Common Pheasant | 1 | 0.017741 | 0.172005 |
| Phoenicurus auroreus | | Daurian Redstart | 4 | 0.028851 | 0.144601 |
| Phoenicurus frontalis | | Blue-fronted Redstart | 5 | 0.079293 | 0.374004 |
| Phoenicurus schisticeps | | White-throated Redstart | 2 | 0.051887 | 0.447047 |
| Prionailurus bengalensis | | Leopard cat | 18 | 0.247137 | 0.785924 |
| Prunella strophiata | | Rufous-breasted Accentor | 7 | 0.229073 | 1.324628 |
| Pseudois nayaur | | Blue Sheep | 40 | 8.009278 | 15.76164 |
| Pucrasia macrolopha | | Koklass Pheasant | 6 | 0.073822 | 0.394456 |
| Pyrrhocorax graculus | | Alpine Chough | 7 | 0.634576 | 4.492703 |
| Pyrrhocorax pyrrhocorax | | Red-billed Chough | 5 | 0.133863 | 0.828402 |
| Rhinopithecus bieti | | Black Snub-nosed Monkey | 1 | 0.006192 | 0.060033 |
| Sus scrofa | | Wild boar | 6 | 0.036039 | 0.138973 |
| Tamiops swinhoei | | Swinhoe's Striped Squirrel | 6 | 0.10281 | 0.533696 |
| Tarsiger chrysaeus | | Golden Bush Robin | 1 | 0.005569 | 0.053998 |
| Tarsiger cyanurus | | Orange-flanked Bluetail | 1 | 0.005454 | 0.052879 |
| Tarsiger rufilatus | | Himalayan Bluetail | 2 | 0.050155 | 0.434809 |
| Tetraogallus tibetanus | | Tibetan Snowcock | 7 | 0.407461 | 2.104449 |
| Tetraophasis szechenyii | | Buff-throated Monal Partridge | 16 | 0.84941 | 3.081786 |
| Trochalopteron elliotii | | Elliot's Laughingthrush | 3 | 0.210092 | 1.542688 |
| Tupaia belangeri | | Northern Treeshrew | 2 | 0.076143 | 0.633853 |
| Turdus kessleri | | Kessler's Thrush | 2 | 0.033217 | 0.270009 |
| Turdus mandarinus | | Chinese Blackbird | 2 | 0.016362 | 0.117737 |
| Turdus rubrocanus | | Chestnut Thrush | 4 | 0.061363 | 0.385475 |
| Ursus thibetanus | | Asiatic black bear | 1 | 0.006192 | 0.060033 |
| Vulpes ferrilata | | Tibetan Fox | 16 | 0.300693 | 1.06166 |
| Yuhina gularis | | Stripe-throated Yuhina | 1 | 0.005569 | 0.053998 |
| Zoothera dixoni | | | 1 | 0.012384 | 0.120066 |

## Slide 2
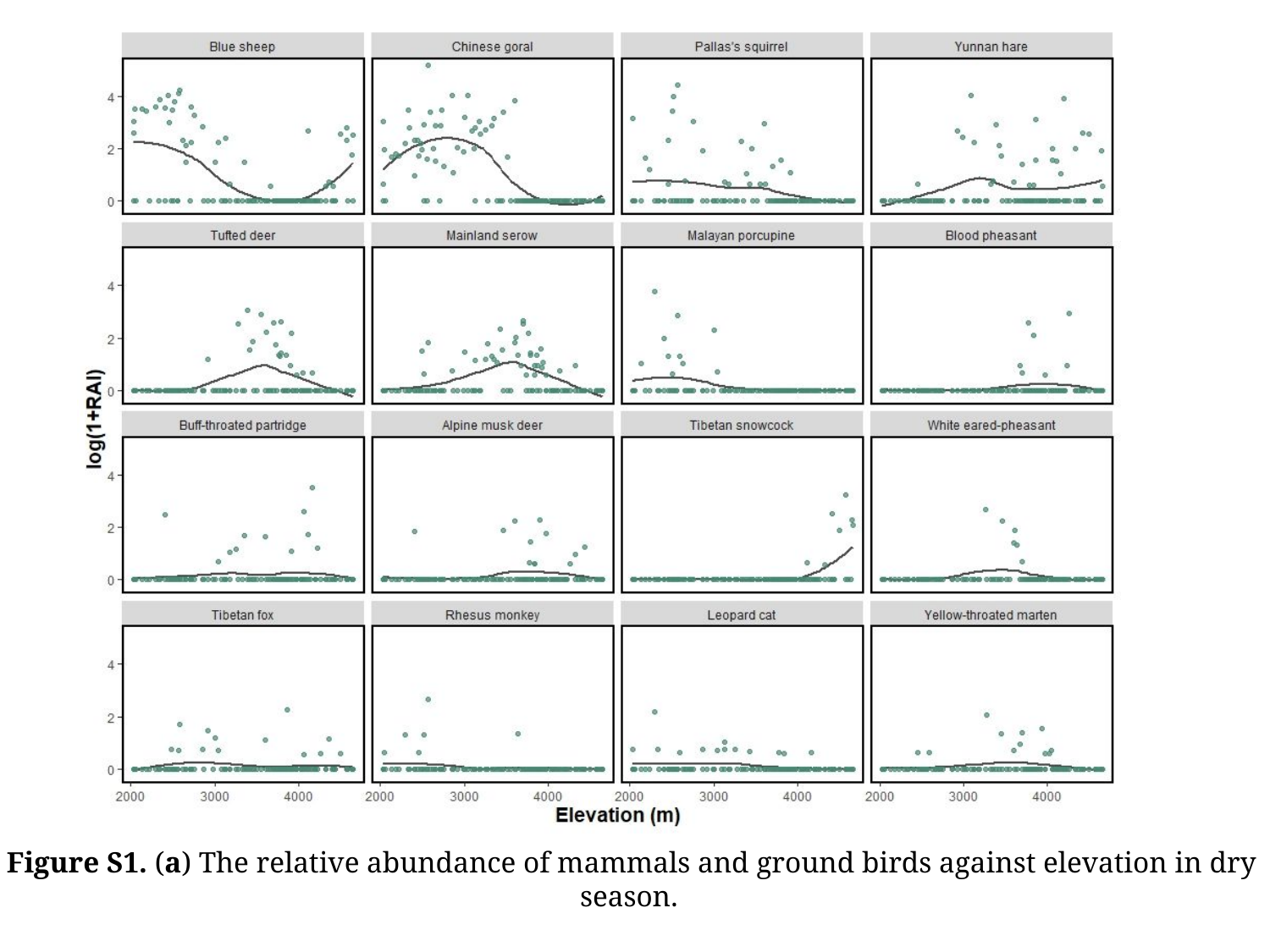

Figure S1. (a) The relative abundance of mammals and ground birds against elevation in dry season.

## Slide 3
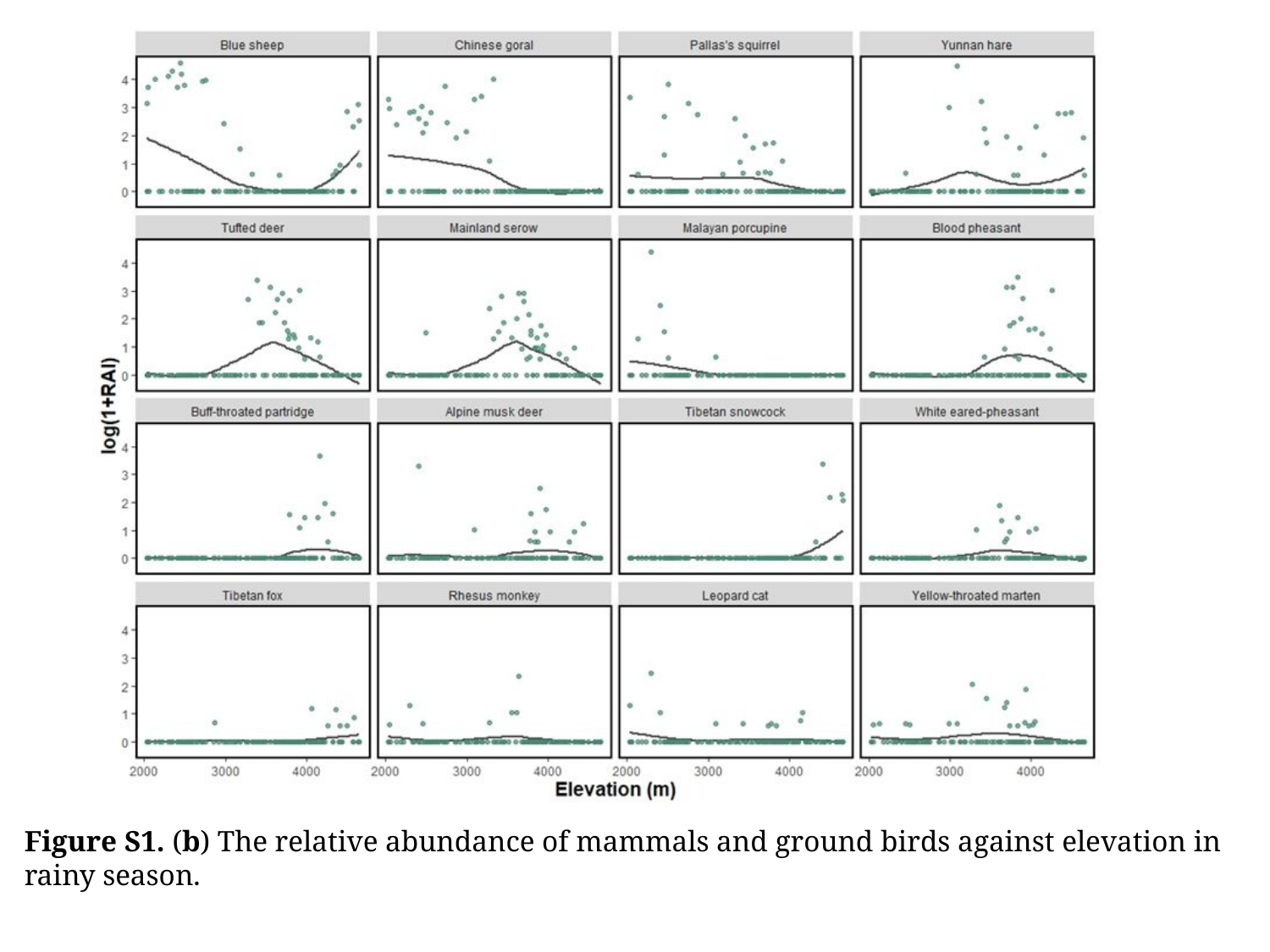

Figure S1. (b) The relative abundance of mammals and ground birds against elevation in rainy season.

## Slide 4
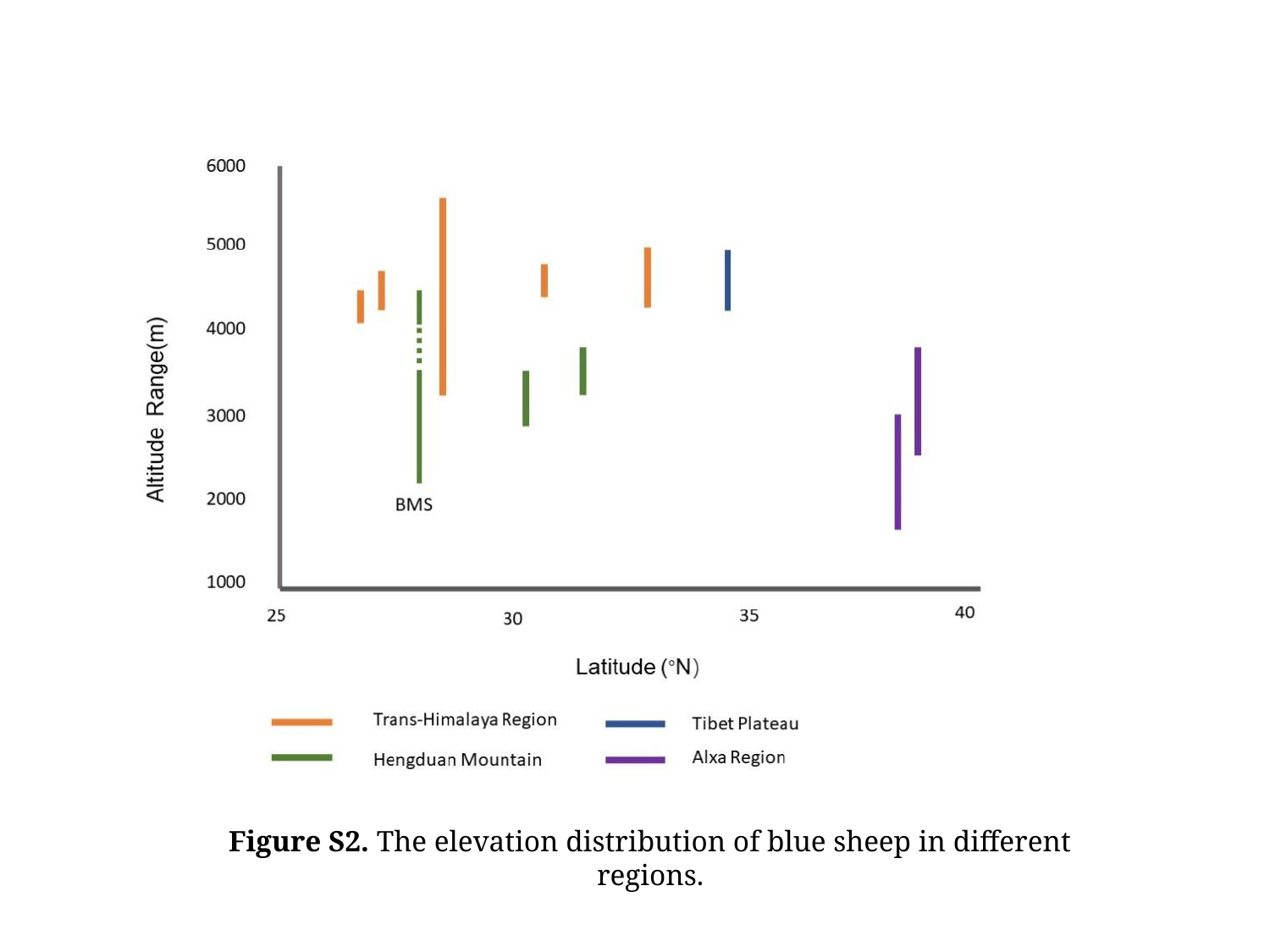

Figure S2. The elevation distribution of blue sheep in different regions.
